# Personal

**Published:** 1901-06

**Authors:** 


### PERSONAL.

Dr. W. Scott Renner, of Buffalo, who has been in Europe for the past three months, has returned and resumed the practice of his specialty, in this city.

Dr. Ralph H. Willse, U. of B., 1900, who served for the past year as house surgeon at the Emergency Hospital, has removed to Westfield, N.Y., to take charge of a private hospital.

---

Dr. James A. Taggart, of Salamanca, has been elected president of the Medical Society of the County of Cattaraugus. Dr. Taggart is one of the rising physicians in the Southern Tier.

---

Dr. John H. Pryor, of Buffalo, was appointed by Governor Odell as a delegate to represent the State of New York in the American Congress of Tuberculosis, held at New York City, May 15 and 16, 1901.

---

Dr. Lorenzo Burrows, Jr., of Buffalo, has been appointed a member of the Buffalo Board of Pension Examining Surgeons, vice Dr. F. W. Abbott, deceased.

---

Dr. Eli Shriver, Jr., U. of B., 1900, who has served as interne of the Sisters of Charity and Emergency Hospitals, has established offices at 198 Spring Street, Buffalo, for the practice of his profession.

---

Dr. Charles Ferdinand Durand has removed to "The Bachelor," No. 50 West Chippewa Street.

---

Dr. Julius Pohlman, of Buffalo, has moved his office and residence from 378 to 404 Franklin Street. Hours: 9 to 1 and 3 to 5.

---

Dr. John A. Miller, of Buffalo, the official chemist of the Pan-American Exposition, has been appointed state chemist under the department of health. Office and laboratory, 44 and 45 Lewis Block, 13½ East Swan Street.

Dr. William T. Griffin, U. of N., '98, who has served since graduation in the Sisters of Charity and Emergency Hospitals, has established himself in private practice, at 232 Van Rensselaer Street, Buffalo.

---

Dr. L. J. McAdam, announces the removal of his office to 453 Ellicott Square, Buffalo. His residence is at Eden Center, N.Y., where he has taken charge of the Fairview Sanitarium—an institution for the treatment of tuberculous and syphilitic diseases. Hours at Ellicott Square, 10 to 12 and 2 to 4. Practice limited to surgery and surgical diseases. Telephone, Seneca 1417.

Dr. Frederick Peterson, of New York City, has been appointed by Governor Odell a member of the State Commission in Lunacy to fill the vacancy caused by the removal of Dr. Peter M. Wise. By virtue of the appointment Dr. Peterson becomes president of the commission and his term is for six years. His many Buffalo friends will be very gratified and the appointment is a most excellent one.

Dr. William A. Brady, U. of B., '01, of Canandaigua, has been appointed a member of the medical staff of Erie County Hospital.

Dr. George S. Peck, of Youngstown, O., announces that hereafter his practice will be limited to surgery, gynecology and consultations.

Dr. S. A. Knopf, of New York, read a paper on The Twentieth Century Problems of the Medical Profession and the Treatment of Tuberculosis, before the section on medicine of the Buffalo Academy of Medicine, Tuesday evening, May 14, 1901. Dr. Julius Ullman invited a group of Buffalo physicians to meet Dr. Knopf at dinner at the Castle Inn just before the meeting.

Dr. H. A. Foster, of Buffalo, has returned from California where he and Mrs. Foster have been spending the winter.

Dr. Julius Ullman and Dr. A. E. Wohnert have been elected chairman and secretary respectively of the section on medicine of the Buffalo Academy of Medicine.

Dr. B. C. Loveland, of Syracuse, has removed his office and residence from 608 S. Salina Street to Fayette Park, 402 E. Genesee Street. Hours: Until 9 a.m.; 2 to 5 p.m. Evenings by appointment. Telephone, 1727 A.

Dr. Maurice J. Lewi, of New York, secretary of the State Board of Medical Examiners, read a paper at the annual meeting of the Medical Society of the County of Westchester, at White Plains, May 21, 1901, entitled, Some Phases of Medical Legislation.

